# The role of NLRP3 inflammasome in psychotropic drug-induced hepatotoxicity

**DOI:** 10.1038/s41420-022-01109-y

**Published:** 2022-07-09

**Authors:** Wenqing Mu, Guang Xu, Ziying Wei, Zhilei Wang, Qin Qin, Li Lin, Lutong Ren, Tingting Liu, Zhie Fang, Yan Yang, Jing Zhao, Junnan Wang, Xiaoyan Zhan, Xiaohe Xiao, Zhaofang Bai

**Affiliations:** 1 Department of Hepatology, the Fifth Medical Center of PLA General Hospital, Beijing, 100039 China; 2 School of Pharmacy, Chengdu University of Traditional Chinese Medicine, Chengdu, 611137 China; 3 School of Traditional Chinese Medicine, Capital Medical University, Beijing, 100069 China; 4 Military Institute of Chinese Materia, Fifth Medical Center of Chinese PLA General Hospital, Beijing, 100039 China; 5 TCM Regulating Metabolic Diseases Key Laboratory of Sichuan Province, Hospital of Chengdu University of Traditional Chinese Medicine, Chengdu, 610072 China

**Keywords:** Cell death and immune response, Hepatotoxicity

## Abstract

Increased medical application of psychotropic drugs raised attention concerning their toxicological effects. In fact, more than 160 psychotropic drugs including antidepressants and antipsychotics, have been shown to cause liver side effects, but the underlying mechanisms are still poorly understood. Here, we discovered that fluoxetine, a common antidepressant, was specifically sensed by NLRP3 inflammasome, whose subsequent activation resulted in the maturation of caspase-1 and IL-1β, as well as gasdermin D (GSDMD) cleavage, which could be completely abrogated by a selective NLRP3 inhibitor MCC950 or *Nlrp3* knockout (*Nlrp3*^*−/−*^). Mechanistically, mitochondrial damage and the subsequent mitochondrial reactive oxygen species (mtROS) accumulation were crucial upstream signaling events in fluoxetine-triggered NLRP3 inflammasome activation. In fluoxetine hepatotoxicity models, mice showed the alterations of aminotransferase levels, hepatic inflammation and hepatocyte death in an NLRP3-dependent manner, and MCC950 pretreatment could reverse these side effects of fluoxetine. Notably, we also found that multiple antidepressants, such as amitriptyline, paroxetine, and imipramine, and antipsychotics, such as asenapine, could specifically trigger the NLRP3 inflammasome activation. Collectively, our findings implicate multiple psychotropic drugs may act as danger signals sensed by the NLRP3 inflammasome and result in hepatic injury.

## Introduction

Drug-induced liver injury (DILI) is widely regarded as a serious health burden. It is a leading reason of failures in drug development and one of the two most frequent causes for drug withdrawals and restrictions [1, 2]. Although it has been reported that the incidence of DILI ranges from 2 per 100,000 to 19 per 100,000 patient-years, many studies show that the risk of this hepatotoxicity is seriously underestimated [3, 4].

Since exploding rates of mental diseases, as well as subsequent increasing application of psychotropic agents, more and more evidences emerge on their liver-toxic effects. Previous studies have shown that more than 160 psychotropic drugs, including antidepressants and antipsychotics, can cause liver-side effects [5]. As the most common psychotropic drugs, almost all antidepressants are generally considered to cause unpredictable, dose-independent liver injury, even at therapeutic doses, and this hepatotoxicity usually develops between several days and six months during drug intake [6]. Antipsychotics, another common psychotropic drugs, also have been shown to be closely associated with the risk of DILI. The common first-generation antipsychotics, like chlorpromazine, and second-generation antipsychotics, such as risperidone and olanzapine, evidence of hepatotoxicity have been provided in both cases and animal studies [7–11]. Simultaneously, it is worth noting that multiple psychotropic drugs are strongly related to adverse metabolic effects such as diabetes and obesity, which further highlighting the need to recognize the hepatotoxic potential of psychotropic drugs [12, 13]. However, the psychotropic drug-induced DILI is a challenging not only in terms of diagnosis, but also in terms of management.

NLRP3 inflammasome, one of the most representative immune multiprotein platforms, is composed of a sensor NLRP3, adapter ASC, and inflammatory protease caspase-1. This platform is activated in response to diverse molecular patterns [14]. Studies have shown that the NLRP3 inflammasome activation requires two sequential signals: a priming signal and an activation signal. After sensing danger signals, the activated caspase-1 triggers maturation and secretion of proinflammatory cytokine IL-1β and, under certain conditions, to induction of gasdermin D (GSDMD)-mediated pyroptosis [15–17]. As an essential component of host defense, however, the dysregulated NLRP3 inflammasome activity causes uncontrolled inflammation, which underlies multiple diseases, such as gout [18], type 2 diabetes [19], atherosclerosis [20], and idiosyncratic drug-induced liver injury (IDILI) [21]. Growing evidences suggest that excessive activation of the NLRP3 inflammasome is a key risk factor for hepatotoxicity [22], and have been reported that application of the NLRP3 inflammasome blockade can reduce hepatic inflammation and fibrosis in mice [23]. Additionally, studies also have shown the role of NLRP3 inflammasome in hepatic injury driven by antiepileptic agents such as carbamazepine [24] and antituberculosis drugs such as isoniazid [25].

In our study, we revealed multiple psychotropic drugs including fluoxetine, asenapine, amitriptyline, paroxetine, and imipramine, could act as danger signals to specifically trigger the NLRP3 inflammasome activation accompanied by caspase-1 maturation, IL-1β secretion and GSDMD cleavage. Mitochondrial damage and the subsequent mitochondrial reactive oxygen species (mtROS) accumulation, which were requirements for the NLRP3 inflammasome activation triggered by fluoxetine. These data demonstrated that the NLRP3 inflammasome may serve as a potential target for the development of novel therapeutics for patients with hepatic injury induced by psychotropic drugs.

## Results

### Multiple psychotropic drugs with the ability to induce DILI trigger the activation of inflammasome

**Fig. 1:**
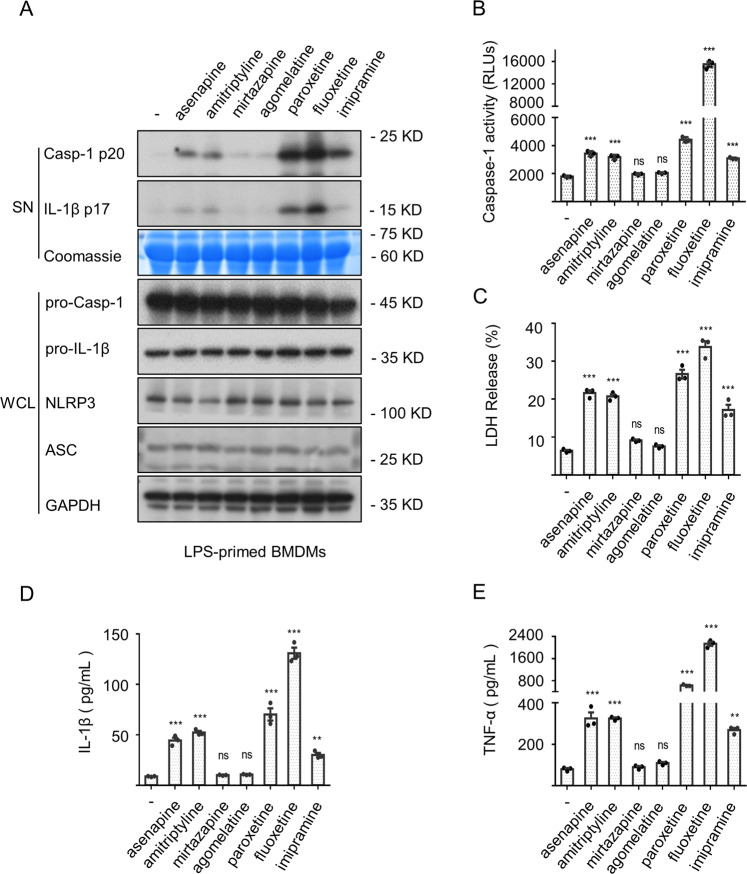
Numerous psychotropic drugs directly trigger the inflammasome activation. **A–E** BMDMs were pretreated with LPS and then treated with these psychotropic drugs for 12 h. Western blots of cell supernatants (SN) and whole-cell lysates (WCL) (**A**) were assessed. The caspase-1 activity (**B**) and the release of LDH (**C**) in SN were detected. The secretion of IL-1β (**D**) or accumulation of TNF-α (**E**) was detected by ELISA. Data are represented as the mean ± SEM from three biological samples, *P < 0.05, **P < 0.01, ***P < 0.001 *vs*. the control. One-Way ANOVA analysis was followed by Dunnett’s post-hoc test (ns, not significant).

Given the potential of psychotropic drugs in driving DILI, we were curious about the role of inflammasome in it. Here, seven common clinical psychotropic drugs (asenapine, amitriptyline, mirtazapine, agomelatine, paroxetine, fluoxetine and imipramine) related to liver injury were chosen for testing. As shown in Fig. 1A, B and D, asenapine, amitriptyline, paroxetine, fluoxetine and imipramine directly triggered the inflammasome activation evidenced by caspase-1 maturation and IL-β generation. Meanwhile, the release of lactate dehydrogenase (LDH) (Fig. 1C) (a programmed cell death associated with inflammation) and the production of an inflammasome-independent cytokine TNF-α (Fig. 1E) were observed. The potential of fluoxetine in inducing the secretion of downstream effector cytokines prompted us to conduct a more in-depth study on it.

### Multiple psychotropic drugs specifically induce the NLRP3 inflammasome activation

**Fig. 2:**
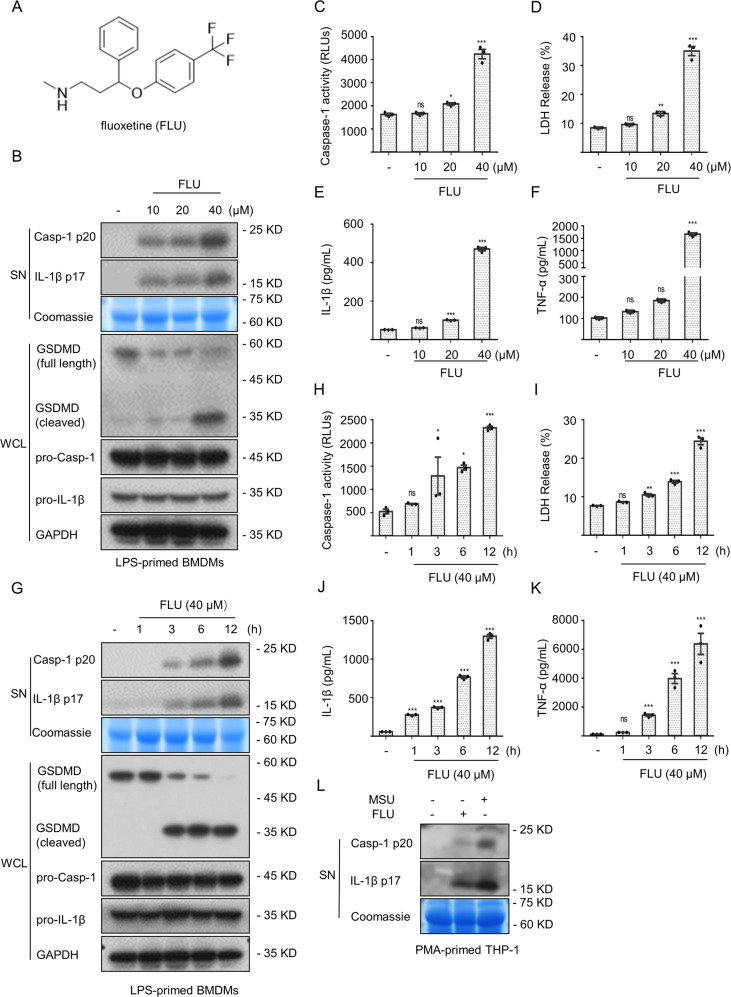
Fluoxetine triggers the maturation of downstream effector cytokines and cleavage of GSDMD. **A** The fluoxetine chemical structure. **B–F** BMDMs were pretreated with LPS and subsequently stimulated with a range of fluoxetine concentrations. The cleaved caspase-1, as well as IL-1β in SN and the cleaved GSDMD in WCL (**B**) were assessed by western blotting. The caspase-1 activity (**C**) and the LDH release (**D**) were measured. Using ELISA to measure the secretion of IL-1β (**E**) and accumulation of TNF-α (**F**) in SN. **G**–**K** BMDMs were first primed with LPS and then stimulated with fluoxetine (40 μM) for 1, 3, 6, and 12 h, respectively. The expression levels of the cleaved caspase-1 and IL-1β in SN and the cleaved GSDMD (**G**) in WCL were detected using western blot analysis. Activity of caspase-1 (**H**), release of LDH (**I**), secretion of IL-1β (**J**) as well as generation of TNF-α (**K**) in SN were also assessed. **L** PMA-primed THP-1 cells were stimulated with fluoxetine and MSU, and then the expression levels of cleaved caspase-1 and IL-1β were detected by western blotting. Data are represented as the mean ± SEM from three biological samples, *P < 0.05, **P < 0.01, ***P < 0.001 *vs*. the control, ns, no significant. One-Way ANOVA analysis was followed by Dunnett’s post-hoc test.

We further investigated the role of fluoxetine (Fig. 2A) on the inflammasome activation. BMDMs were pretreated with LPS followed by fluoxetine stimulation. The results indicated that the production of caspase-1 and IL-β, and cleavage of GSDMD were triggered by fluoxetine in a dose-dependent manner (Fig. 2B, C and E). Similarly, the release of LDH (Fig. 2D) and the production of TNF-α (Fig. 2F) were associated with the dose of fluoxetine. Meanwhile, LPS-primed BMDMs were stimulated with fluoxetine for 1, 3, 6, and 12 h, respectively, and we observed the caspase-1 maturation (Fig. 2G and H), GSDMD cleavage, IL-β generation (Fig. 2G and J), LDH release (Fig. 2I), and TNF-α generation (Fig. 2K) in a time-dependent manner. Furthermore, THP-1 cells were pretreated with PMA for 4 h followed by fluoxetine and MSU stimulation for 12 h. As shown in Fig. 2L, both of fluoxetine and MSU could trigger the maturation of caspase-1 and IL-1β.

**Fig. 3:**
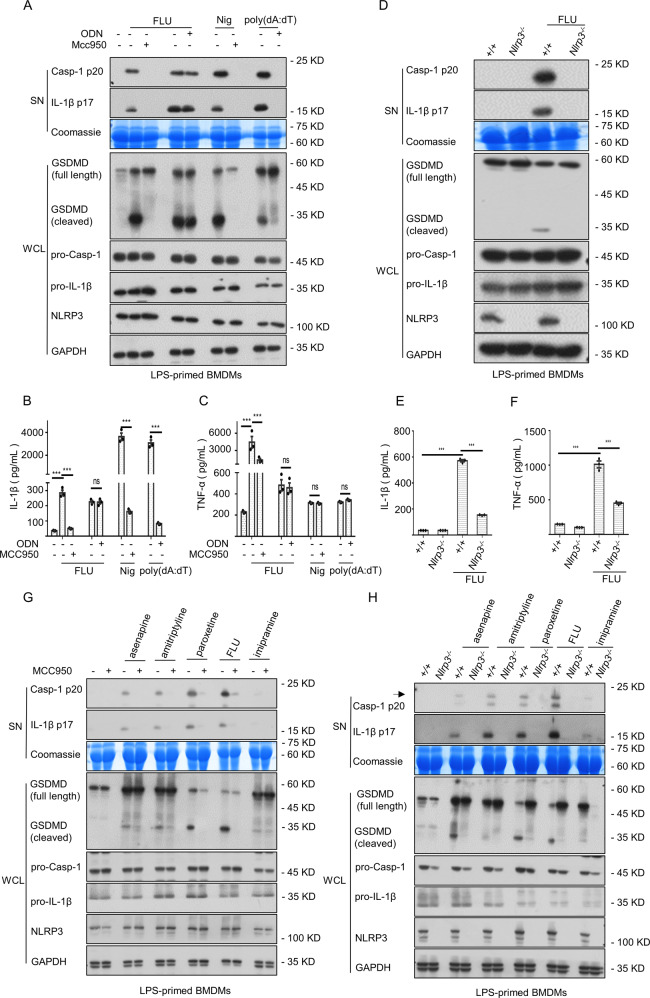
Multiple psychotropic drugs specifically induce NLRP3 inflammasome activation. **A**–**C** In the presence or absence of ODN or MCC950, fluoxetine, Nig as well as poly(dA:dT) were incubated in LPS-primed BMDMs. The maturation of caspase-1, secretion of IL-1β in SN and the cleavage of GSDMD in WCL (**A**) were assessed by western blotting. Using ELISA kits to test the levels of IL-1β (**B**) and TNF-α (**C**) in SN. **D**–**F** WT and *Nlrp3*^*−/−*^ BMDMs were preated with LPS, and then stimulated with fluoxetine. Western blotting analysis was applicated to assess the expression levels of caspase-1 and IL-1β in SN and the cleavage of GSDMD (**D**) in WCL. The levels of IL-1β (**E**) and TNF-α (**F**) in SN were measured by ELISA kits. **G** LPS-primed BMDMs were pretreated with MCC950 and then stimulated with asenapine, amitriptyline, paroxetine, fluoxetine and imipramine, the expression of cleaved caspase-1 and IL-1β in SN and cleaved GSDMD in WCL were assessed by western blotting. **H** WT and *Nlrp3*^*−/−*^ BMDMs were pretreated with LPS, and then stimulated with these psychotropic drugs. Western blotting analysis was applicated to assess the cleaved caspase-1 and IL-1β in SN and the cleaved GSDMD in WCL. Data are represented as the mean ± SEM from three biological samples; ***P < 0.001, ns, not significant, unpaired Student’s t test (two groups) or One-Way ANOVA analysis followed by Dunnett’s post-hoc test (multi groups).

Additionally, NLRP3 has the extraordinary capacity of sensing damage-associated molecular patterns (DAMPs) such as MSU, ATP, or silica [26]. Since both antidepressant fluoxetine and MSU could directly trigger inflammasome activation, we wondered whether the generation of inflammatory cytokines induced by fluoxetine would also occur through the NLRP3 inflammasome. To elucidate this, BMDMs were treated with LPS to provide for the activation of NLRP3 inflammasome by inducing the synthesis of NLRP3 and pro-IL-1β, and then stimulated with fluoxetine, nigericin (Nig), and poly(dA:dT). The fluoxetine-induced caspase-1 maturation, IL-1β generation, GSDMD cleavage, and TNF-α accumulation were abrogated in the presence of MCC950 (a specific NLRP3 inflammasome blocker) [27] (Fig. 3A–C). In contrast, AIM2 inflammasome did not appear to be essential for fluoxetine signaling, because the administration of synthetic oligodeoxynucleotide (ODN) [28], a selective and potent blocker of AIM2, completely blocked poly(dA:dT)-triggered AIM2 inflammasome activation rather than fluoxetine (Fig. 3A–C). To further clarify the requirement for the NLRP3 inflammasome in antidepressant fluoxetine-induced downstream events. We then assessed the effect of fluoxetine on inflammasome activation in *Nlrp3* knockout (*Nlrp3*^*−/−*^) BMDMs. As shown in Fig. 3D and E, the fluoxetine-triggered the maturation of caspase-1 and IL-1β were completely abrogated in *Nlrp3*^*−/−*^ BMDMs. Furthermore, the cleavage of GSDMD and production of TNF-α were also blocked in *Nlrp3*^*−/−*^ BMDMs (Fig. 3D and F).

Notably, we also found that other psychotropic drugs including asenapine, amitriptyline, paroxetine, and imipramine could trigger inflammasome-dependent cytokines caspase-1 and IL-1β production, and cleavage of GSDMD. Moreover, they could be abrogated by MCC950 pretreatment or *Nlrp3* deficiency (Fig. 3G and H, Fig. S1A and B). Likewise, there was an obvious influence on TNF-α production (Fig. S1C and D). Taken together, multiple psychotropic drugs could specifically trigger NLRP3 inflammasome activation to induce the secretion of downstream effector cytokines.

### Mitochondrial damage and mtROS accumulation are crucial molecular signals in fluoxetine-induced NLRP3 inflammasome activation

**Fig. 4:**
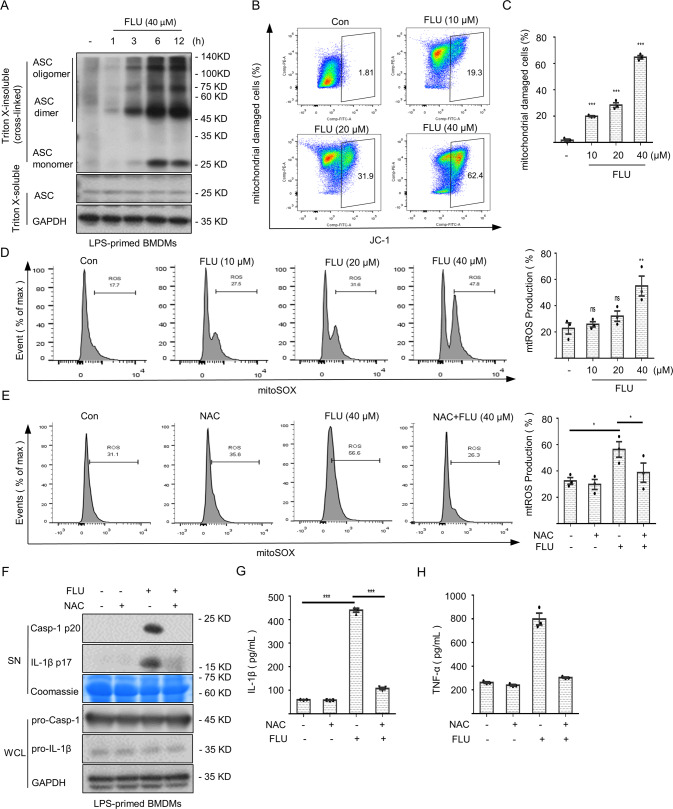
Mitochondrial damage and mtROS accumulation are crucial molecular signals for fluoxetine-induced NLRP3 inflammasome activation. **A** The oligomerization of ASC after DSS crosslinking in WCL was measured by western blotting. **B**, **C** The mitochondrial damage was assessed using a JC-1 mitochondrial membrane potential assay kit. **D** LPS-primed BMDMs were stimulated with fluoxetine for 6 h and the mtROS content was measured by flow cytometry. **E**–**H** LPS-primed BMDMs were pretreated with NAC and then treated with fluoxetine (40 μM) for 6 h, the mtROS content (**E**) was measured by flow cytometry. The expression levels of cleaved caspase-1 and IL-1β in SN (**F**) were detected by western blotting and using ELISA kits to detect the secretion of IL-1β (**G**) and generation of TNF-α (**H**) in SN. Data are shown as the mean ± SEM from three biological samples; *P < 0.05, **P < 0.01, ***P < 0.001, ns, not significant. One-Way ANOVA analysis followed by Dunnett’s post hoc test.

Next, we further studied how fluoxetine triggered NLRP3 inflammasome activation. ASC oligomerization seems to be an indispensable step in NLRP3 inflammasome activation [29], offering conditions for the secretion of mature cytokines. As shown in Fig. 4A, fluoxetine triggered dimerization as well as oligomerization of the ASC molecules in a time dependent manner, suggesting that fluoxetine acted upstream events to induce the activation of NLRP3 inflammasome.

The mitochondrial damage and release of the mtROS into cytosol are essential upstream signaling events implicated in the NLRP3 inflammasome activation [30, 31]. Then, we assessed the role of mitochondrial damage in fluoxetine-induced the NLRP3 inflammasome activation. JC-1 staining results indicated that fluoxetine directly triggered mitochondrial damage (Fig. 4B and C). Furthermore, we also wondered whether the accumulation of mtROS was involved in fluoxetine-induced the aberrant activation of the NLRP3 inflammasome. To clarify this, BMDMs were treated with LPS followed by fluoxetine stimulation. The amount of mtROS accumulation was detected by flow cytometry. The results indicated that fluoxetine could induce mtROS generation in a dose-dependent manner (Fig. 4D). We then pretreated LPS-primed BMDMs with N-Acetylcysteine (NAC, a ROS scavenger) [32] to further verify whether the mtROS affected the fluoxetine-triggered NLRP3 inflammasome activation. As shown in Fig. 4E, the accumulation of mtROS was inhibited. Meanwhile, fluoxetine-induced production of caspase-1 and IL-1β were indeed impaired in response to the NAC (Fig. 4F and G). Similarly, the decrease of TNF-α was also observed (Fig. 4H). In summary, these data suggested that the mitochondrial damage and the subsequent mtROS accumulation were indispensable steps in fluoxetine-induced NLRP3 inflammasome activation.

### Fluoxetine induces idiosyncratic hepatotoxicity through NLRP3 inflammasome

**Fig. 5:**
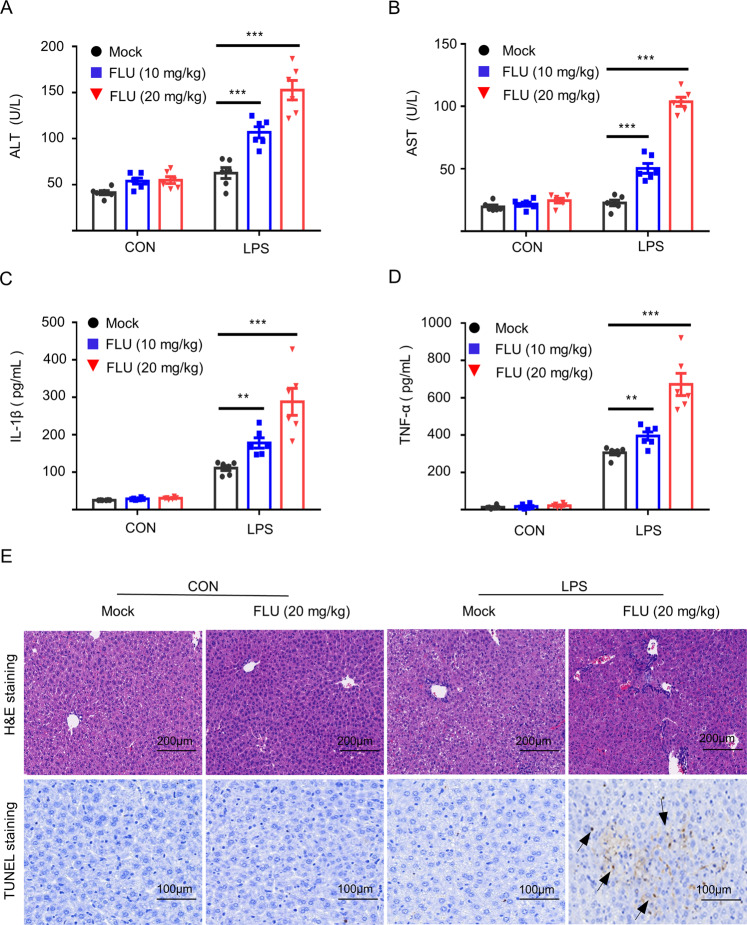
Fluoxetine induces hepatic injury via triggering the activation of the NLRP3 inflammasome in vivo. **A**–**D** WT C57BL/6 mice were injected with LPS (2 mg/kg) and then stimulated with fluoxetine (10 mg/kg, 20 mg/kg, *n* = 6/group). The levels of mouse serum ALT (**A**) and AST (**B**) were measured by GTP and GOT kits, and IL-1β (**C**) and TNF-α (**D**) were detected by ELISA kits. **E** H&E staining (scale bar: 200 μm) and TUNEL staining (scale bar: 100 μm) were used to assess inflammatory infiltration and TUNEL positive. Data are shown as the mean ± SEM. *P < 0.05, **P < 0.01, ***P < 0.001vs LPS group, ns, not significant. One-Way ANOVA analysis followed by Dunnett’s post-hoc test.

Previous studies have reported that fluoxetine could induce unpredictable, dose-independent hepatotoxicity [33]. Additionally, among the coexisting inflammation factors, LPS is generally considered to be a determinant of susceptibility to IDILI [34]. Here, we simulated IDILI in mice through co-exposing to non-hepatotoxic doses of LPS and fluoxetine, and assessed the role of NLRP3 inflammasome in fluoxetine-driven hepatic injury in vivo. Animals were treated with LPS and subsequently stimulated with fluoxetine. As shown in Fig. 5A–D, fluoxetine alone did not result in any pathological changes compared with the control group. However, compared with other groups, combination of fluoxetine and LPS induced elevation of the levels of serum AST, ALT, IL-1β, and TNF-α. Additionally, TUNEL staining and H&E staining showed that co-exposure to LPS and fluoxetine induced liver inflammation and hepatocyte focal necrosis, which were not occurred in the fluoxetine group and control group (Fig. 5E). Collectively, these data indicate that for the development of idiosyncratic hepatotoxicity in fluoxetine using individuals requires participation of additional inflammation inducers such as LPS. Thus, likelihood of idiosyncratic hepatotoxicity development in fluoxetine-using individuals is higher when they have inflammation-related diseases such as bacterial infection or gout.

### Fluoxetine-induced idiosyncratic hepatic injury could be reversed by MCC950 pretreatment in vivo

**Fig. 6:**
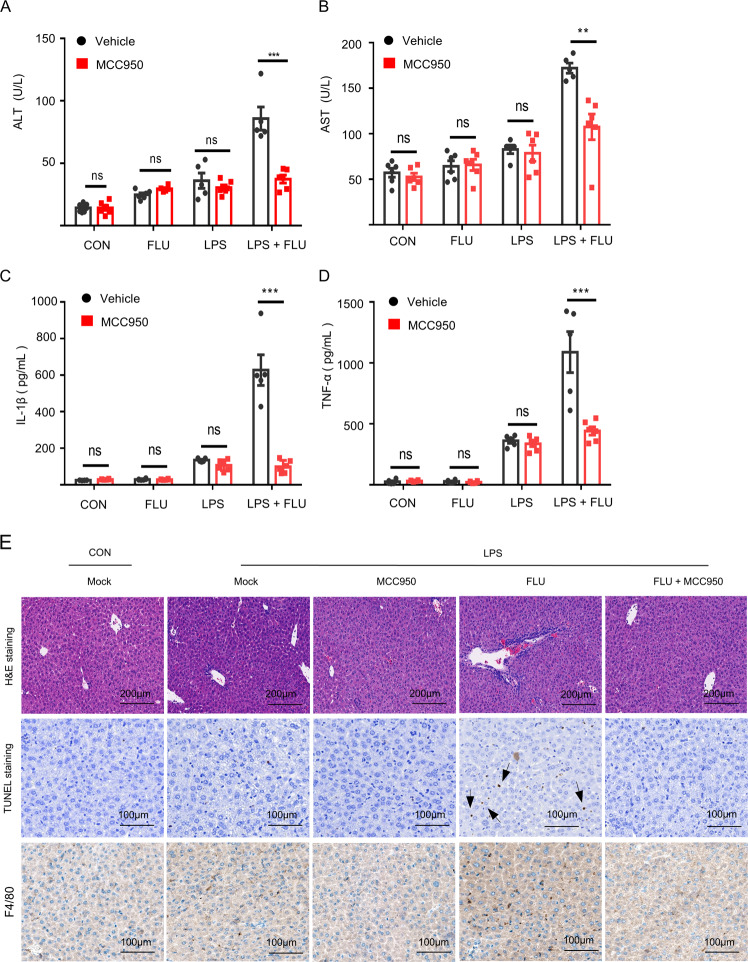
MCC950 pretreatment rescues LPS/fluoxetine-induced hepatotoxicity. **A**–**D** WT C57BL/6 mice were pretreatment with MCC950 (50 mg/kg, *n* = 5 LPS and LPS/FLU groups, *n* = 6 other groups) and then treated with LPS (2 mg/kg) and finally fluoxetine (20 mg/kg) was administrated. The levels of mouse serum ALT (**A**), AST (**B**), IL-1β (**C**), and TNF-α (**D**) were assessed by corresponding kits. **E** H&E staining (scale bar: 200 μm), TUNEL staining (scale bar: 100 μm) and F4/80 staining (scale bar: 100 μm) were used to analyze the liver inflammatory infiltration and TUNEL positive. Data are shown as the mean ± SEM. ***P < 0.001 *vs* the LPS/FLU group, ns, not significant, Statistics differences were analyzed using Student’s t test.

Next, the MCC950 were administered before mice were co-exposed to LPS and fluoxetine, to block the NLRP3 inflammasome activation in vivo. According to the biochemical analysis, we found that pretreatment with MCC950 could dramatically reduce the serum levels of ALT and AST, and suppress the IL-1β production and TNF-a accumulation triggered by LPS/fluoxetine in vivo (Fig. 6A–D). Similarly, the liver inflammation and hepatocyte focal necrosis were observed in the LPS/fluoxetine group through TUNEL staining, H&E staining and F4/80 staining, while these situations were obviously improved in the presence of MCC950 (Fig. 6E). These data suggested that the NLRP3 inflammasome played a crucial role in fluoxetine/LPS-driven hepatotoxicity.

## Discussion

The psychotropic drug-induced hepatotoxicity is a serious health threat and one of the leading reasons for limiting their development. In our study, the psychotropic drugs fluoxetine [35], asenapine [36], amitriptyline [37], paroxetine [38], and imipramine [39] with hepatotoxicity reports specifically triggered the NLRP3 inflammasome activation accompanied by the secretion of downstream effector cytokine such as IL-1β, as well as the production of the indirectly regulated cytokine TNF-α. We thought that the production of TNF-α has been indirectly regulated by inflammasome activation, because the inflammasome-dependent inflammatory cytokine IL-1β could induce TNF-α production by activating NF-κB signaling pathway [40, 41]. What supported our inference was that our study Fig. 3C and F showed that the production of TNF-α induced by fluoxetine could be blocked by the selective NLRP3 inhibitor MCC950 pretreatment or *Nlrp3* deficiency.

Additionally, the fluoxetine hepatotoxicity models showed that fluoxetine alone could not cause hepatic injury in the absence of LPS. However, in the presence of LPS, fluoxetine induced alterations in liver enzyme levels, hepatic inflammation and hepatocyte death, and this is mediated by the NLRP3 inflammasome. These results suggested that the IDILI related to aberrant activation of the NLRP3 inflammasome caused by fluoxetine may depend on a mild inflammatory state induced by LPS. Consistent with our studies, it has been reported that coexisting inflammatory mediators like LPS could be regarded as the determinants of susceptibility to IDILI [34] and non-hepatotoxic doses of LPS could decrease the threshold for toxicity and/or increase the magnitude of response [42]. Furthermore, given that other psychotropic drugs could also specifically trigger the NLRP3 inflammasome activation, the role of NLRP3 inflammasome in psychotropic drug-driven hepatotoxicity should be considered. Meanwhile, the model of fluoxetine hepatotoxicity has revealed that MCC950 pretreatment could reverse the hepatic injury when co-exposure to LPS and fluoxetine, suggesting that these adverse reactions caused by multiple psychotropic drugs could be rescued or prevented by small molecule inhibitors of the NLRP3 inflammasome.

As studies reported, multiple DAMPs such as monosodium urate (MSU) crystals [43], cholesterol crystals [44], asbestos, and silica [26, 45] are usually regarded as the NLRP3 inflammasome agonist. Meanwhile, they drive various inflammatory-related diseases such as gout [43], atherosclerosis [46], silicosis [26], and even DILI [47] by inducing aberrant activation of the NLRP3 inflammasome. Consistent with these studies, our findings showed that multiple psychotropic drugs directly triggered aberrant activation of the NLRP3 inflammasome, indicating that in certain special cases, they can be regarded as exogenous DAMPs, triggering aberrant response of the innate immune and resulting in idiosyncratic hepatotoxicity.

Interestingly, the effects of selective serotonin reuptake inhibitors (SSRIs) on inflammatory response are contradictory since these agents act either as anti- or pro-inflammatory. As the most common SSRI, fluoxetine has been reported to play an anti- or proinflammatory role in microglia related to the quality of the living environment [48]. Additionally, it is worth noting that the response of fluoxetine to inflammation in different tissues is also paradoxical. For example, fluoxetine affects depression by inhibiting the activation of NLRP3 inflammasome in microglia [49]. Furthermore, fluoxetine mitigates NLRP3 inflammasome and caspase-1 activation through autophagy activation after subarachnoid hemorrhage (SAH) to treat early brain injury [50]. In contrast, fluoxetine treatment shows a marked pro-inflammatory effect in liver tissue. The previous study has shown that fluoxetine treatment can lead to liver inflammation and oxidative stress [33]. Other studies also have shown that fluoxetine induces portal zone inflammation, lobular inflammation. and hepatomegaly [51, 52]. Consistent with these studies, our work suggest that fluoxetine induces idiosyncratic hepatotoxicity by triggering aberrant activation of the NLRP3 inflammasome. Although we still poorly understood the underlying molecular mechanisms of fluoxetine’s differential response in inflammation, monitoring of potential liver injury during fluoxetine treatment appears to be warranted.

In summary, our studies demonstrate that fluoxetine and other psychotropic drugs with similar effects can act as exogenous DAMPs directly trigger the NLRP3 inflammasome activation accompanied by caspase-1 activation, IL-1β secretion and GSDMD mediated pyroptosis. Meanwhile, mitochondria damage and mtROS accumulation as the crucial molecular signals that are conducive to fluoxetine triggere its activation. In this way, combined with selective small molecule inhibitors of NLRP3 inflammasome may be a valid therapeutic strategy for the treatment of liver injury caused by multiple psychotropic drugs.

## Materials and Methods

### Mice

Wild-type (WT) female C57BL/6 mice (eight-week-old) in the study were obtained from SPF Biotechnology Co., Ltd of Beijing, China. Female *Nlrp3*^*−/−*^ mice were supported by Dr. Tao Li from National Center of Biomedical Analysis (Beijing, China). All mice were maintained under a pathogen-free condition (22 ± 2 °C) and held under a 12-h dark/light cycle. We tried our best to minimize the suffering as well as the number of animals used. When assessing experimental outcomes, the investigators were blinded to the treatments.

### Chemicals and antibodies

The chemicals and antibodies in our study were listed in Supplemental Table 1. All small molecular compounds were dissolved in DMSO.

### Cell preparation and culture

BMDMs were isolated and collected from the *Nlrp3*^*−/−*^ mice or WT mice using a standard reverse perfusion procedure and then cultured in Dulbecco’s modified Eagle’s medium (DMEM) containing 10% fetal bovine serum (FBS), 1% penicillin/streptomycin, as well as murine recombinant mouse macrophage colony-stimulating factor (MCS-F; 50 ng/mL). Additionally, human THP-1 cells, which were a gift from Dr. Tao Li from NCBA, were incubated in Roswell Park Memorial Institute (RPMI) 1640 medium (1% penicillin/streptomycin and 10% FBS) and primed with 100 nmol PMA for 6 h. Cells were kept in a humidified 5% (v/v) CO2 incubator at 37 °C.

### Inflammasome activation assay

To assess the activation of the inflammasome, WT and/or *Nlrp3*^*−/−*^ BMDMs (1.2 ×10^6^ cells/mL) were incubated in 12-well plates overnight. Next, cells were pretreated with ultrapure LPS (50 ng/mL) for 4 h and then stimulated with various psychotropic drugs (asenapine, amitriptyline, mirtazapine, agomelatine, paroxetine, fluoxetine and imipramine, 40 μM) for 12 h, respectively. The inflammasome-related downstream effector cytokines were detected.

### Caspase assay

Activity of caspase-1 in cell supernatants was measured by Caspase-Glo 1 reagent. The experimental protocol of activity of the caspase-1 assay was similar to described previously [53, 54].

### Immunoblotting

After psychotropic drugs treatment, the cell supernatants were collected and then trichloroacetic acid was added to precipitate proteins. The method of western blotting analysis has been described previously [55].

### LDH release

BMDMs were pretreated with LPS and then treated with psychotropic drugs for twelve hours. Then, the release of LDH in cell culture supernatants was detected using a LDH cytotoxicity assay kit.

### Enzyme-linked immunosorbent assay (ELISA)

The levels of TNF-α and IL-1β in cell supernatants and serum were measured using Mouse ELISA Kits, which were performed under the manufacturer’s instructions.

### ASC oligomerization

LPS-primed BMDMs were stimulated with fluoxetine (40 μM) for 1, 3, 6, and 12 h, respectively, and then lysed with Triton Buffer [56] for 15 min. Next, these samples were collected and centrifuged at 6500 g for 15 min (4 °C). The supernatants were used as whole-cell lysates (WCL) and the pellet were washed and resuspended with 200 μL phosphate-buffered saline (PBS) and then cross-linked with 4 mM disuccinimidyl suberate (DSS) at 37 °C for 30 min with rotation. After centrifuging at 6500 g for 15 min, the cross-linked pellets were collected and resuspended in 1 x SDS loading buffer for western blotting assay.

### Measurement of mitochondrial damage and mtROS measurement

The JC-1 mitochondrial membrane potential assay kits were used to assess the damage of mitochondrial. BMDMs in diameter culture dish tubes (100 mm) were pretreated with LPS for four hours. Then, the cells were transferred to 1.5 mL tubes and stimulated with fluoxetine for 6 h and then incubated with JC-1 (10 μM) at 37 °C for fifteen minutes. After cells were washed with PBS twice and resuspended with 200 μL PBS, flow cytometry was used to evaluate the mitochondrial damage. Similarly, to assess the fluoxetine-induced mtROS accumulation, BMDMs in the diameter culture dish tubes were treated with LPS and then transferred to tubes followed by fluoxetine stimulation for six hours. Next, the samples were washed with hank’s balanced salt solution (HBSS) twice and stained with 4 mM MitoSOX Red mitochondrial superoxide dismutase indicator for fifteen minutes. After staining and washing, 200 μL HBSS were added to resuspend cells and the production of mtROS was conducted by flow cytometry.

### Biochemical analysis

The mouse serum levels of AST and ALT were evaluated with commercially available kits (Nanjing Jiancheng Bioengineering Institute).

### Stimulation with fluoxetine in vivo

LPS (2 mg/kg) or PBS vehicles was administered to the WT C57BL/6 mice (female; 8-week-old; *n* = 6/group) *via* tail vein for two hours. Then, the mice were injected intraperitoneally with fluoxetine (10 mg/kg or 20 mg/kg) or its carrier (PBS) six hours before mouse serum and liver tissue were collected. The serum levels of ALT or AST was measured with GPT or GOT kits and the serum levels of TNF-α or IL-1β was assessed with the Mouse ELISA kits. Simultaneously, using hematoxylin and eosin (H&E) staining and TUNEL staining to evaluate the injury of liver tissue.

### Combination of MCC950 and fluoxetine

The WT C57BL/6 mice (female; 8-week-old; *n* = 5 LPS and LPS/FLU groups, *n* = 6 other groups) were injected intraperitoneally with 50 mg/kg MCC950 or PBS. One hour later, LPS (2 mg/kg) or its carrier were given via the tail vein and then fluoxetine (20 mg/kg) or PBS were administrated via intraperitoneal injection. After 6 h, the mouse serum levels of ALT, AST, IL-1β, and TNF-α were detected by corresponding kits, and the liver tissues injury were assessed by H&E staining, TUNEL staining and F4/80 staining.

### Statistical analyses

Excel was used for statistics and GraphPad Prism 6 (a GraphPad Software) was applicated for analysis. All of the results were presented as the mean ± SEM. Comparisons were performed by One-Way ANOVA analysis (multigroups) or Student’s t-test (two groups). The difference was considered statistically significant when *P* < 0.05.

## Supplementary information

Supplementary table 1

Supplementary figure legends

Supplementary figure 1

Original images

## Data Availability

All data will be made available upon request.
