# Supplementary material for: The role of NLRP3 inflammasome in psychotropic drug-induced hepatotoxicity
Source: Cell Death Discov. 2022 Jul 9;8:313. doi: 10.1038/s41420-022-01109-y (PMC9271040; doi:10.1038/s41420-022-01109-y)
Supplement: Supplementary file 1 — Supplementary table 1 [file 41420_2022_1109_MOESM1_ESM.docx]

**Supplemental Table 1.**

**KEY RESOURCES TABLE**

| **REAGENT or RESOURCE** | **IDENTIFIER** | **SOURCE** | **REGION** |
| --- | --- | --- | --- |
| Dulbecco’s modified Eagle’s medium (DMEM) | CM10013 | MACGENE | Beijing, China |
| 1640 medium | CM10040 | MACGENE | Beijing, China |
| fetal bovine serum (FBS) | 04-001-1ACS | Gibco | New York, USA |
| mouse macrophage colony stimulating factor (MCSF) | HY-P7085 | MedChemExpress (MCE) | NJ, USA |
| Opti-MEM | 31985-670 | Gibco | New York, USA |
| Ultrapure LPS | tlrl-pb5lps | InvivoGen | San Diego, USA |
| MCC950 | HY-12815A | MCE | NJ, USA |
| asenapine | HY-10121 | MCE | NJ, USA |
| protriptyline | HY-B0949 | MCE | NJ, USA |
| amitriptyline | HY-B0527A | MCE | NJ, USA |
| mirtazapine | HY-B0352 | MCE | NJ, USA |
| agomelatine | [HY-17038](https://www.medchemexpress.cn/Agomelatine.html) | MCE | NJ, USA |
| paroxetine | HY-B0492 | MCE | NJ, USA |
| fluoxetine | HY-B0102A | MCE | NJ, USA |
| imipramine | HY-B1490 | MCE | NJ, USA |
| synthetic oligodeoxynucleotide | tlrl-ttag151 | InvivoGen | San Diego, USA |
| N-Acetylcysteine | HY-B0215 | MCE | NJ, USA |
| PMA | HY-18739 | MCE | NJ, USA |
| MSU | Tlrl-msu-25 | InvivoGen | San Diego, USA |
| JC-1 Mitochondrial Membrane Potential Assay Kit | HY-K0601 | MCE | NJ, USA |
| MitoSOX^TM^ Red mitochondrial supperoxide indicator | 2311845 | Invitrogen | Carlsbad, CA, USA |
| anti-mouse Caspase-1 | AG-20B-0042-C100 | Adipogen | San Diego, USA |
| anti-NLRP3 | AG-20B-0014-C100 |  |  |
| anti-mouse IL-1β | AF-401-NA | R&D | Minnesota, USA |
| Mouse-IL-1β | SMLB00C |  |  |
| disuccinimidyl suberate (DSS) | ab141274 | abcam | Cambridge, UK |
| anti-mouse GSDMD | ab209845 |  |  |
| anti-ASC | A1170 | ABclonal | Wuhan, China |
| anti-GAPDH | 60004-1-1g | Proteintech | Chicago, USA |
| anti-DDDDK tag | 0543-1-AP | Proteintech | Chicago, USA |
| anti Myc-NLRP3 | 60003-2-1g | Proteintech | Chicago, USA |
| Mouse IL-1β | 1210122 | DAKEWE | Beijing, China |
| Mouse-TNF-α | 1217202 | DAKEWE | Beijing, China |
| GPT | C009-2-1 | Nanjing Jiancheng Bioengineering Institute | Nanjing, China |
| GOT | C010-2-1 |  |  |
