# Supplementary material for: The role of NLRP3 inflammasome in psychotropic drug-induced hepatotoxicity
Source: Cell Death Discov. 2022 Jul 9;8:313. doi: 10.1038/s41420-022-01109-y (PMC9271040; doi:10.1038/s41420-022-01109-y)
Supplement: Supplementary file 2 — Supplementary figure legends [file 41420_2022_1109_MOESM2_ESM.docx]

**Supplementary figure legends**

**Fig. S1. Multiple** **Psychotropic drugs specifically trigger NLRP3 inflammasome activation. (**A, C) LPS-primed BMDMs were pretreated with MCC950 and then stimulated with psychotropic drugs. The levels of IL-1β (A) and TNF-α (C) in cell culture supernatants (SN) were detected by ELISA kits. (B, D) WT and *Nlrp3^-/-^* BMDMs were primed with LPS and then stimulated with the psychotropic drugs. ELISA kits were applicated to detect the levels of IL-1β (B) and TNF-α (D) in SN. Data are expressed as the mean ± SEM, (n=3); **P < 0.01, ***P < 0.001 .vs the control group; unpaired Student’s *t-*test.
