# Supplementary figures and images for: The role of NLRP3 inflammasome in psychotropic drug-induced hepatotoxicity
Source: Cell Death Discov. 2022 Jul 9;8:313. doi: 10.1038/s41420-022-01109-y (PMC9271040; doi:10.1038/s41420-022-01109-y)

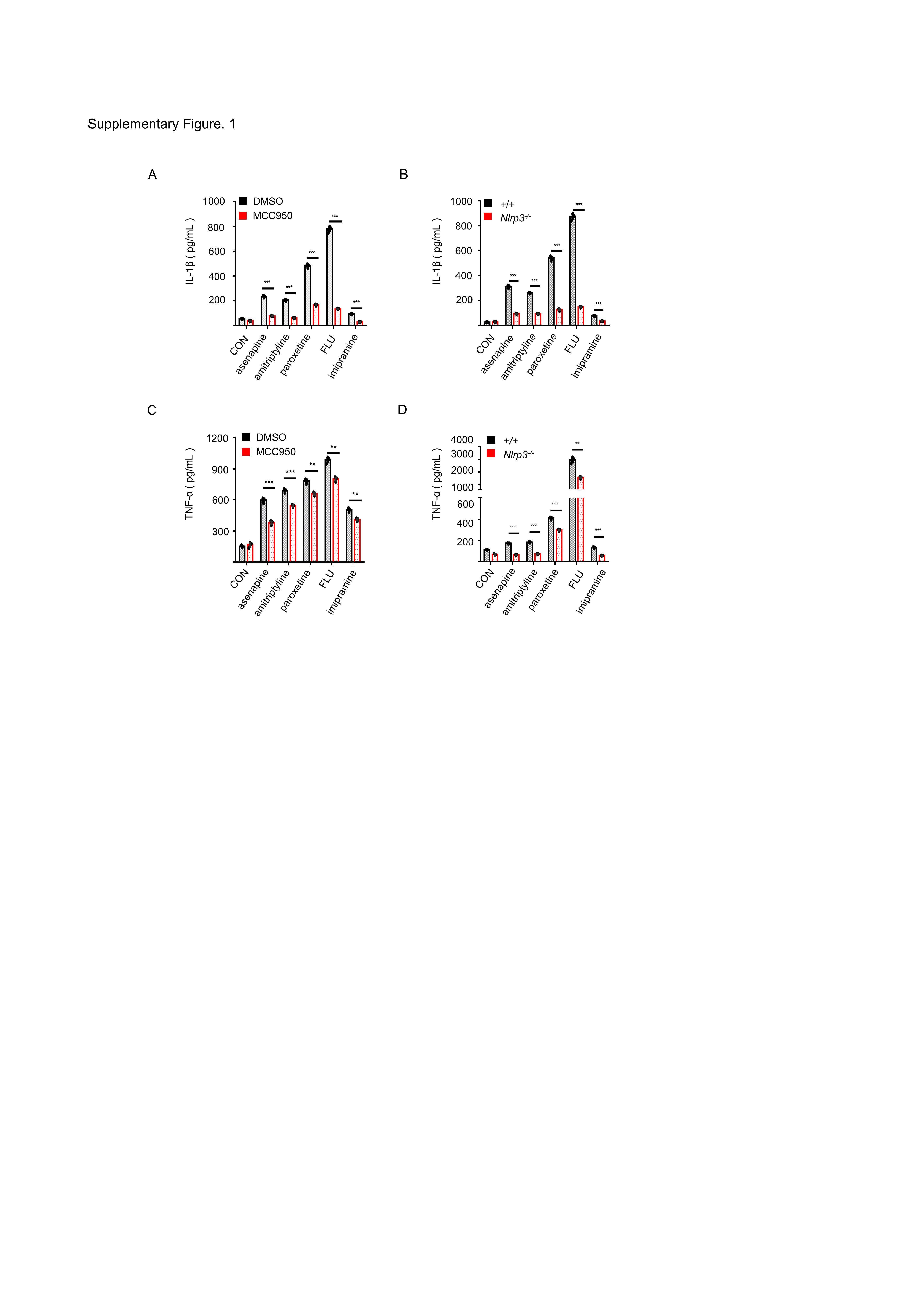

Supplement: Supplementary file 3 — Supplementary figure 1 [file 41420_2022_1109_MOESM3_ESM.tif]

**Original western blots**


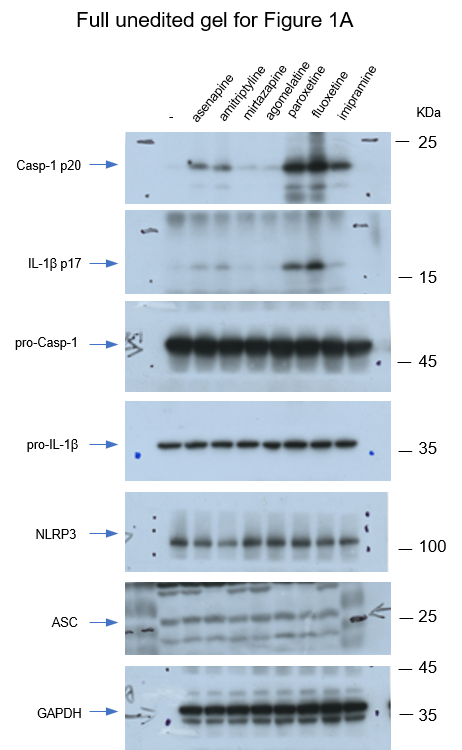


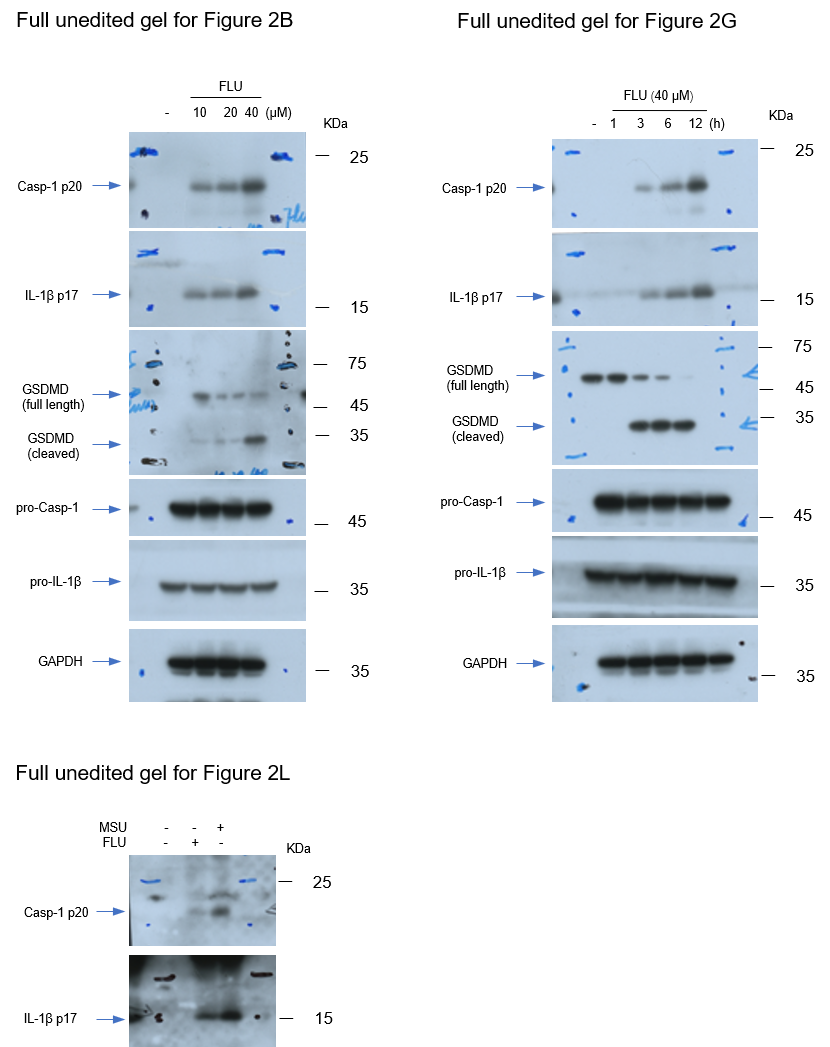


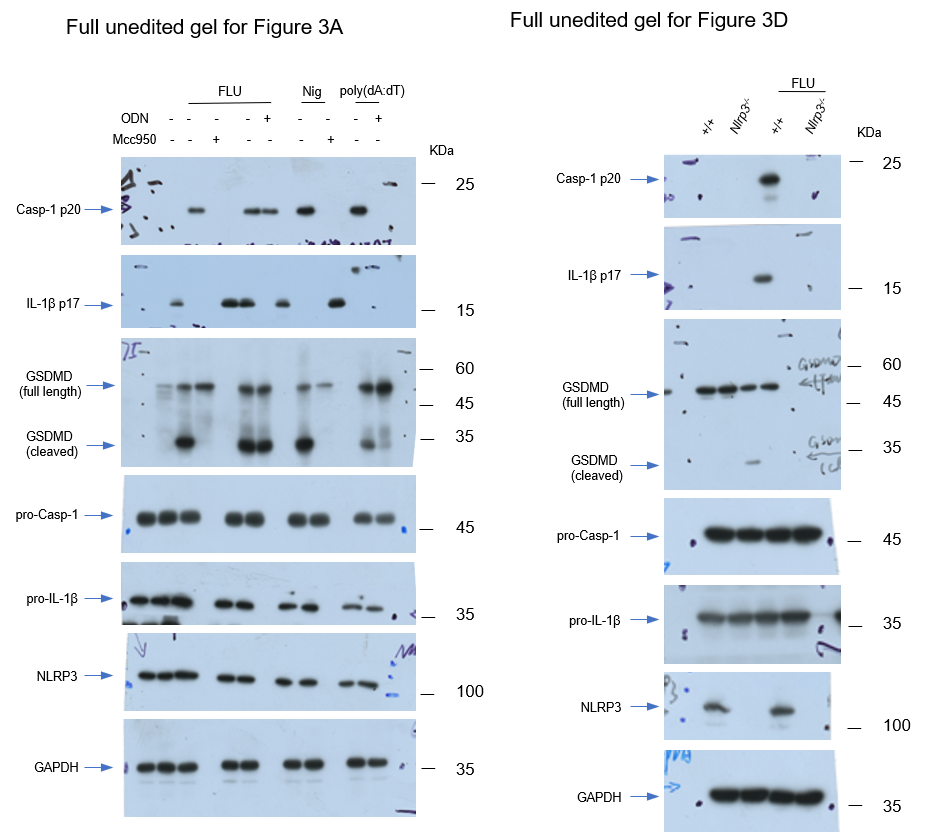


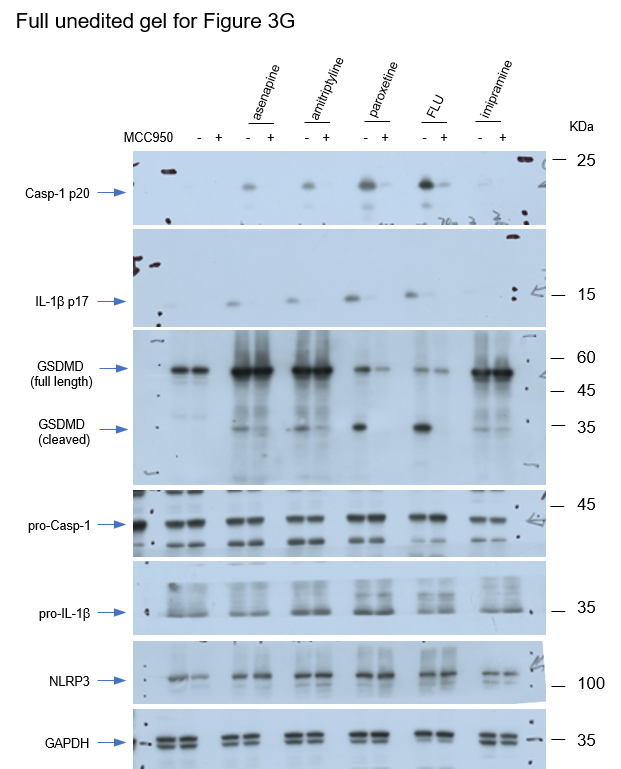


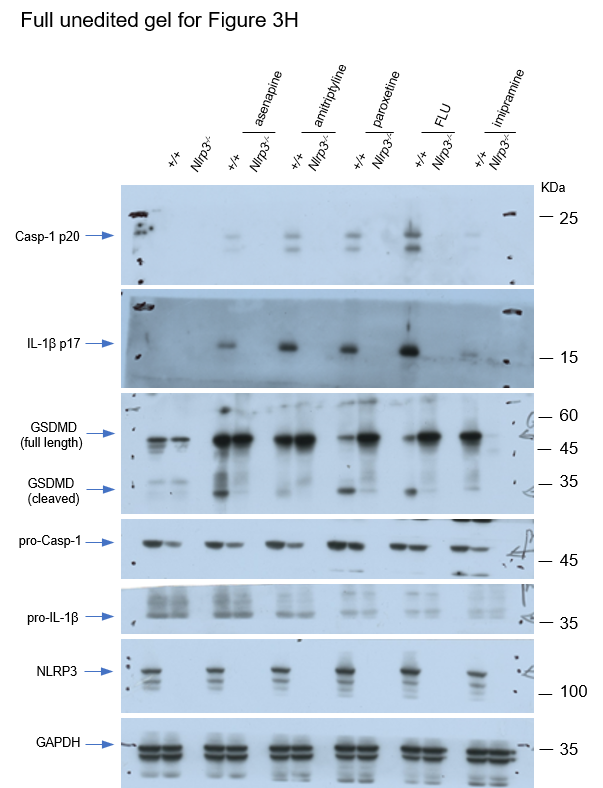


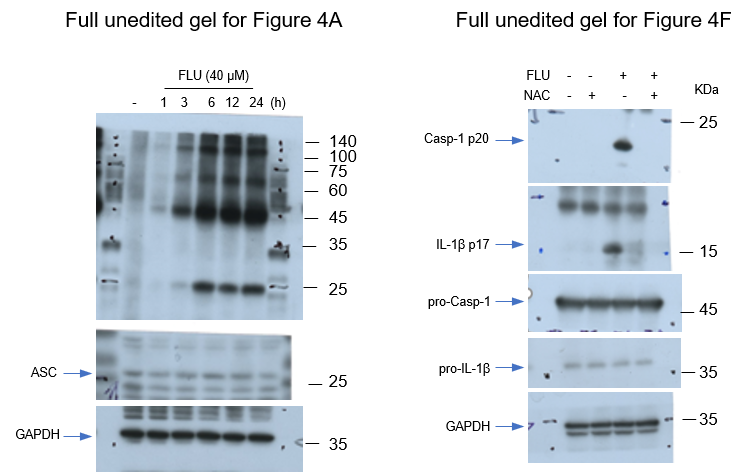

Supplement: Supplementary file 4 — Original images [file 41420_2022_1109_MOESM4_ESM.docx]
